# Use of exogenous fibrolytic enzymes and probiotic in finely ground starters to improve calf performance

**DOI:** 10.1038/s41598-022-16070-0

**Published:** 2022-07-13

**Authors:** A. R. Khademi, F. Hashemzadeh, M. Khorvash, A. H. Mahdavi, A. Pazoki, M. H. Ghaffari

**Affiliations:** 1 Department of Animal Sciences, College of Agriculture, Isfahan University of Technology, Isfahan, 84156–83111 Iran; 2 Ghiam Agriculture and Animal Husbandry, Isfahan, 83145–46600 Iran; 3 Institute of Animal Science, University of Bonn, 53111 Bonn, Germany

**Keywords:** Developmental biology, Physiology

## Abstract

The present study investigated the effects of adding wheat straw treated with exogenous fibrolytic enzymes (EFE) and a probiotic supplement to finely ground starters on growth performance, rumen fermentation, behavior, digestibility, and health of dairy calves. A total of 48 Holstein dairy calves (39.8 ± 1.67 kg body weight) were randomly assigned to one of 4 nutritional treatments (n = 12 calves per treatment). The experiment was conducted in a 2 × 2 factorial arrangement of treatments consisting of two diets with or without EFE-treated wheat straw (2 g/day/calf) and diets with or without probiotics (2 g/day/calf). All calves were weaned on day 63 and remained in the study until day 84. The addition of EFE to wheat straw had no effect on starter feed intake, increased neutral detergent fiber (NDF) digestibility and recumbency, but decreased average daily gain (ADG) after weaning (240 g/d). The addition of probiotics to the diet had no effect on starter feed intake, improved feed efficiency, ADG (150 g/d), final weight (11.3 kg), and NDF digestibility, and decreased the ratio of acetate to propionate in the rumen. The addition of probiotics to starter feed for calves could improve their growth.

## Introduction

The ultimate goal of nutritionists is to maximize calf starter intake and rumen development prior to weaning. Current feeding strategies for young calves are based on starter diets high in non-structural carbohydrates that promote rumen microbiota establishment and short-chain fatty acid (SCFA) production and subsequently support rumen papillae development^1^, which has long-term effects on growth, health, and milk yield in adult cattle. However, some studies have clarified that excessive intake of rapidly fermentable carbohydrates can also predispose calves to rumen acidosis^2^, which can lead to cancerous proliferation and keratinization of rumen epithelial cells^3^ and impairs rumen SCFA uptake^4^. The addition of dietary fiber to starter feed for calves may prevent the epithelium from building up a keratin layer^5^, especially if the particle size or texture of the starter feed is poor^6^.

It has been demonstrated that the addition of fibrolytic enzymes to the diet can improve weight gain and feed conversion in steers or calves by increasing nutrient digestibility^7^. Fibrolytic microorganisms in the rumen take a longer time to establish in the rumen than starch- and sugar-fermenting microorganisms^8^, which may reduce digestion of fibrous material and limit utilization efficiency^9^. Therefore, the addition of fibrolytic enzymes to starter diets containing low digestibility forages is expected to promote digestibility and improve performance in young calves. In support, the addition of fibrolytic enzymes has been reported to have a positive effect on SCFA production in the rumen^10^ and to increase populations of *Fibrobacter succinogenes* and *Butyrivibrio fibrisolvens* in the rumen, resulting in improved performance of calves during the pre- and post-weaning periods^11^. In addition, Wang et al.^11^ found that supplementation with fibrolytic enzymes increased microbial enzyme activities in the rumen after weaning, including carboxymethylcellulase, cellobiase, xylanase, and pectinase, compared with calves without supplementation. Both supplements, fibrolytic enzymes and probiotics, have the potential to modulate rumen fermentation and improve fiber digestibility, animal performance, and feed conversion efficiency. Some researchers have investigated the synergistic interaction between these two additives in in vitro and in vivo studies^12–14^. Despite conflicting results in in vitro studies, Malik and Bandla^12^ reported that the combination of probiotics and fibrolytic enzymes had a reciprocal beneficial effect on rumen fermentation and improved growth performance and feed efficiency of growing buffalo calves.

Young dairy calves can be fed probiotics in a starter feed or liquid feed to promote solid feed intake and support gut health and improve growth performance^15^. The most significant effects of probiotic supplementation in the preweaning period were reported when administered to calves during periods of stress^15^. Probiotics promote optimal maturation of pathogenic colonization^16^. Villot et al.^17^ reported that calves fed *Saccharomyces cerevisiae boulardii* in milk replacer, a subspecies of *Saccharomyces cerevisiae*, showed no decrease in daily weight gain during diarrheal disease compared with control calves, which experienced a large decrease in growth performance during diarrheal disease. In young calves, the addition of direct-fed microbiome to starter feed resulted in decreased lactate production and improved rumen pH, although total viable and cellulolytic bacteria increased by 33.5 and 57.4%, respectively^18^. Le et al.^19^ demonstrated that supplementation of *Bacillus amyloliquefaciens* strain H57 promoted rumen development, as evidenced by increased plasma β-hydroxybutyrate concentrations and improved nutritional performance and health status of dairy calves during the transition from milk to dry feed. However, little information is available on how the addition of exogenous fibrolytic enzymes (EFE) may affect calf growth performance before and after weaning.

We hypothesized that the addition of EFE and probiotics would mitigate the adverse effects of feeding dairy calves rapidly degradable carbohydrates from finely ground starters and improve rumen fermentation, nutrient digestibility, and growth performance. Therefore, the objective of our study was to investigate the effects of EFE-treated wheat straw and the addition of probiotics to finely ground starters on growth performance, rumen fermentation, behavior, digestibility, and health of dairy calves before and after weaning.

## Materials and methods

### Animals, management, and treatments

The study was conducted at a local commercial dairy farm (FKA Animal Husbandry and Agriculture Co., Isfahan, Iran). Ethical approval for all procedures involving animals was obtained from the Animal Care and Use Committee of Isfahan University of Technology (IUT, Iran; IACUC #2020/A05) prior to the start of the study. All methods were performed according to the regulations of the Iranian Council of Animal Care (1995). The study complies with the ARRIVE guidelines for reporting in vivo experiments, and all methods were performed in accordance with the relevant guidelines and regulations.

In this dairy farm, calves were separated from their dams immediately after birth, weighed, and housed in a single pen. The quality of the colostrum was measured with a digital Brix refractometer (PAL -1, Atago Co. Ltd.) and discarded if it had a value less than 21 on the Brix scale. All calves received 4 l of good-quality pasteurized colostrum (Brix values ≥ 22%) with nipple bottles 1 h after birth and an additional 2 l of colostrum 6 h after the first feeding. Calves that had difficulty sucking received colostrum either via an esophageal tube. Blood samples were collected 24 h after the first colostrum feeding by venipuncture from the jugular vein with Clot Activator Vacutainers (BD Vacutainer, Franklin Lakes, NJ), and serum total protein was determined with a commercial refractometer (VET 360; Reichert Inc., Depew, NY). Only calves with a serum protein level > 5.7 mg/dL were included in the study. Calves were fed 4 L of transition milk from day 2 to 3 of life. From day 4 of life, pasteurized whole milk with 3.20 ± 0.15% fat, 3.11 ± 0.08% CP, 5.21 ± 0.09% lactose, and 12.0 ± 0.15% total solids. Calves received 4 l of whole milk from day 4 to 15, 5 l from day 15 to 18, 6 kg from day 18 to 22, and 7 l from day 22 to 60 in two equal meals at 08:00 and 16:00. From day 61 to 62, calves received 3.5 l of milk in the morning feeding. All calves were weaned on day 63 and remained in the study until day 84. During the study, calves were housed in individual outdoor pens (2.9 m × 1.1 m × 1.8 m; length × width × height). Fresh shaving wood was used as bedding and refreshed daily, and manure was removed daily to keep the pens visibly clean and dry.

In a 2 × 2 factorial arrangement of treatments, a total of 48 female Holstein dairy calves (39.8 ± 1.67 kg of BW) were randomly assigned to 1 of 4 dietary treatments (n = 12 calves per treatment). Treatments were (1) calves fed diets without EFE-treated wheat straw and probiotic supplement (EFE-Pro−), (2) calves fed EFE-treated wheat straw but with probiotic supplement (2 g/d/calf; EFE-Pro+), (3) calves fed EFE-treated wheat straw (2 g/day/calf) but without probiotic supplement (EFE + Pro−), (4) calves fed EFE-treated wheat straw and probiotic supplement (EFE + Pro+). Exogenous fibrolytic enzymes were added to the water. Then, wheat straw was added to the water and reconstituted (with tap water) 24 h before feeding by adding the required amount of dry wheat straw to an industrial container (Iran Plast Co., Isfahan, Iran) and mixing thoroughly (every 6 h for a period of 24 h). The container was kept at room temperature in the shade. In this study, a commercially produced probiotic and EFE were used. The probiotic contained *saccharomyces cerevisiae* (10^8^ CFU/g), *Lactobacillus acidophilus* (10^8^ CFU/g), *Lactobacillus plantarum* (10^8^ CFU/g), *Lactobacillus rhamnosus* (10^8^ CFU/g), *Lactobacillus casei* (10^8^ CFU/g), *Bifidobacterium bifidium* (10^8^ CFU/g), *pediococcus acidilactici* (10^8^ CFU/g), *Bacillus subtilis* (10^9^ CFU/g) and *Enterococcus faecium* (10^8^ CFU/g). Fibrolytic enzyme contained Cellulase (6 × 10^6^ u/kg) and Xylanase (1 × 10^7^ u/kg). No milk refusal was observed in any of the treatments. All calves had free access to water and starter feed throughout the study. The diet offered was adjusted daily to achieve a 5% to 10% orts (i.e., the portion of the starter that was not consumed over a 24-h period); orts were collected and weighed at 0800 h daily. Calves were fed according to FKA Animal Husbandry and Agriculture Co recommendations and protocols, and sick calves were treated accordingly by a veterinarian. All experimental diets were formulated according to the Cornell Net Carbohydrate and Protein System, version 5.1 (CNCPS; Table 1). Calves were fed a starter diet containing 7% chopped wheat straw as a TMR with concentrate throughout the study.

**Table 1:** Experimental diets ingredients and chemical composition.

Ingredient, %	Experimental starter feeds	Chemical composition	EFE+		EFE−	
			Pro+	PRO−	Pro+	PRO−
Wheat straw	7	DM, %	85.1	84.6	84.2	84.6
Ground barley	5.48	OM, %	91.4	91.7	91.5	90.7
Ground corn	43	CP, %	20.8	20.9	20.8	20.8
Soybean meal	25.9	EE, %	3.7	3.5	3.6	3.6
Fish meal	4.65	NDF, %	13.9	14.4	16.9	16.8
Calcium carbonate	1.47	ADF, %	8	7.8	7.9	8.1
Dicalcium phosphate	0.28	NFC,^b^ %	53	53.2	50.1	49.4
Sodium bentonite	0.46	ME,^c^ Mcal/kg	2.96	2.96	2.92	2.92
Monensin	0.05	NE_g,_^c^ Mcal/kg	1.35	1.34	1.32	1.32
Salt	0.35					
Mineral and vitamin premix^a^	0.2					

^a^Contained per kilogram of supplement: CP: 290 g; EE: 65 g; ME: 9.66 MJ; NE_G_: 3.56 MJ; NDF: 175 g; Ca: 6.50 g; P: 7.70 g; non-fiber carbohydrate (NFC): 290 g; Mg: 2 g; K: 0.99 g; Na: 1.6 g; Cl: 0.1 mg; Co: 23 mg; Mn: 43 mg; Se: 0.1 mg; Zn: 43 mg; Vitamin A: 12,000 IU; Vitamin D: 5,000 IU; Vitamin E: 100 IU.

^b^Calculated as [DM − (NDF + CP + ether extract + ash)] (NRC, 2001).

^c^Estimated using NRC (2001) equations with the values from the analyses for starter.

### Data collection and sampling

Throughout the study, feed refusers were removed daily just before fresh feed was provided in the morning. Individual feed intake was determined daily by weighing the amounts of feed offered and refused. In addition, representative samples (n = 10) of the feed and orts were taken from each calf twice a month and stored frozen (− 20° C) for later analysis. Subsamples of the dried feed and orts were thoroughly mixed and ground in a mill (Ogaw Seiki Co. Ltd.) so that they passed a 1-mm sieve before chemical analysis. Standard methods described in AOAC international^20^, were used for determination of dry matter (DM, method 2001.12), ash (method 942.05), crude protein (CP, method 991.20), and ether extract (method 920.39). Amylase-treated neutral detergent fiber (aNDF) and acid detergent fiber (ADF) were analyzed by the Ankom220 Fiber Analyzer (Macedon, New York, United States) using heat-stable α-amylase (100 μL/0.5 g of sample) and sodium sulfite, which was used in the NDF method, according to the procedure described by Van Soest et al.^21^.

Calves were weighed at birth, 3 days after birth, and then every 10 days until the end of the experiment before morning milk feeding using a digital scale [with an electronic scale (model EES-500; Ettehad Inc., Isfahan, Iran) calibrated by the manufacturer's representative before the start of the study and every month thereafter]. Average daily gain (ADG; kg/d) and feed efficiency [FE, kg BW gain/total DMI (DMI = milk DM + starter feed DM)] were calculated every 10 days. Skeletal growth parameters, including body length (the distance between the shoulder points and the rump), withers height (the distance between the base of the front feet and the withers), heart girth (the circumference of the chest), body depth (the circumference of the abdomen before feeding), hip height (the distance between the base of the hind feet and the hook bones), and hip width (the distance between the points of the hook bones) of the calves were measured manually on days 4, 63, and 80 of the study as described by Pazoki et al.^22^. Measurements were taken by an individual who had high interobserver reliability for each measurement.

To determine the total tract apparent digestibility, fecal samples were collected at 14:00 on 5 consecutive days, from day 63 to 67 of calf age. The dry matter (DM) of feed, ort, and fecal samples was determined after they were dried at 65 °C for 48 h and then ground through a 1-mm sieve (Wiley Mill, Arthur Thomas Co., Philadelphia, Pennsylvania, United States). The acid-insoluble ash content of feed and feces was used as an internal marker for calculating the apparent digestibility of the entire digestive tract of DM and organic matter (OM), as described by Van Keulen and Young^23^. Milk samples were collected every two weeks, preserved with potassium dichromate, stored at 4 °C, and then analyzed for concentrations of fat, protein, lactose, and total solids using Milkoscan (Foss Electric, Hillerød, Denmark; AOAC International).

Rumen fluid was collected 4 h after morning feeding with a stomach tube connected to a vacuum pump on days 35 and 70 of the study; the first 100 ml were discarded to avoid contamination with saliva. The rumen contents were squeezed through 4 layers of cheesecloth. 4 mL of the rumen fluid was acidified with 1 mL of 25% metaphosphoric acid and run for analysis for SCFA by gas chromatography (model CP-9002; Chrompack, Middelburg, The Netherlands) which was performed with a 50-m (0.32 mm i.d.) silica-fused column (CP-Wax Chrompack Capillary Column; Varian Medical Systems, Palo Alto, California, United States), with crotonic acid (1:7, vol/vol) as the internal standard, as described by Hashemzadeh-Cigari et al.^24^.

Behavioral parameters [eating, rumination, resting, drinking, standing, lying, and non-nutritive oral behavior (NNOB)] of the calves were visually observed by two trained individuals who were unaware of the treatments for three consecutive days before weaning (d 40 to 43 of the experiment) and after weaning (d 65 to 68 of the experiment) from 08:00 to 16:00 h under daylight conditions. Calves were observed every 5 min and any activity was assumed to continue during the 5-min interval between observations (Kargar et al.^25,26^).

Fecal score was determined daily at 0700 h based on fecal consistency using the procedure of the University of Wisconsin Madison (https://www.vetmed.wisc.edu/fapm/svm-dairy-apps/calf-health-scorer-chs/) as follows: Fecal score: 0 = normal, 1 = semi-formed, pasty, 2 = loose but remains on top of bedding, and 3 = watery, sifts through bedding. General appearance was also observed daily and scored as described by Heinrichs et al.^27^: 1 = normal and alert, 2 = drooping ears, 3 = head and ears drooping, dull eyes, slightly lethargic, 4 = head and ears drooping, dull eyes, lethargic, 5 = severely lethargic. Rectal temperature was measured daily using a digital thermometer (Qingdao Dacon Trading Co. Ltd., Shandong, China). Calves with a fecal score ≥ 2 were classified as having diarrhea. All sick calves were diagnosed according to the standard operating procedures of FKA Agriculture and Animal Husbandry Facility (Isfahan, Iran) and treated with standard procedures prescribed by the veterinarian.

### Statistical analysis

For the primary response variables, including starter intake, BW, and ADG, a power analysis was conducted to estimate sample size^28,29^) based on previous literature values^30–32^. From the power test analysis with α = 0.05 and power = 0.80, the predicted sample size was 12 calves per treatment for starter intake, total DMI, ADG, and BW. All data were tested for normal distribution using the UNIVARIATE procedure of SAS version 9.4 (Shapiro–Wilk test; SAS Institute Inc), and variables that were not normally distributed were log-transformed (base 10) to meet assumptions of normality and homoscedasticity of residuals. Statistical analyzes were performed over 3 time periods: before weaning (4 to 63 days), after weaning (64 to 84 days), and over the entire period (4 to 84 days). Data were analyzed as a completely randomized design with a 2 × 2 factorial arrangement of treatments with EFE and probiotic supplementation as factors. Data were analyzed by repeated measures ANOVA using the PROC MIXED procedure of SAS for starter feed intake, ADG, BW, ADG, FE, structural growth, rumen fermentation, and behavior. The model included the fixed effects of probiotic, EFE, time (day or week) as repeated measures and their interactions. Calf was included as a random effect. Initial BW (day 4) and structural growth measurements were included as covariates for BW and skeletal growth analysis. Four variance–covariance structures (heterogeneous autoregressive type 1, autoregressive type 1, compound symmetry, and Toeplitz) were tested, and the heterogeneous autoregressive type 1 covariance structure was determined to be the most appropriate covariance structure for all repeated statements according to the Akaike and Bayesian information criteria. The main effects of EFE and probiotic supplementation and their interactions were tested using ANOVA. The effects of nutritional treatment on categorical responses related to fecal and health scores were analyzed using the GLIMMIX procedure of SAS version 9.4. The significance threshold was set at *P* ≤ 0.05; trends were reported at 0.05 < *P* ≤ 0.10.

### Principal component analysis

PCA was applied to obtain an overview of growth performance, health and behavioral data based on the different treatments. The MetaboAnalyst 5.0 web-based metabolomics data processing tool was used for this purpose (see http://www.metaboanalyst.ca for detailed methodology)^33^. Data were transformed using the generalized log transformation and then scaled by Pareto (mean-cantered and divided by the square root of the standard deviation of each variable) to correct for heteroscedasticity, reduce skewness, and mask effects.

## Results

### Intake and growth performance

**Figure 1:**
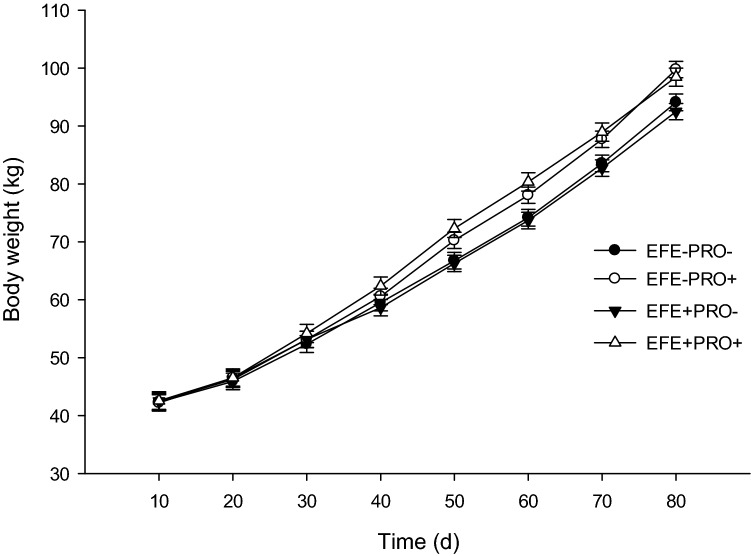
Body weight in calves receiving different experimental diets. Treatments were (1) calves fed diets without exogenous fibrolytic enzymes (EFE)-treated wheat straw and probiotic supplement (EFE-Pro−), (2) calves fed diets of EFE-treated wheat straw but with probiotic supplement (2 g/d/calf; EFE-Pro+), (3) calves fed EFE-treated wheat straw (2 g/d/calf) but without probiotic supplement (EFE+ Pro−), (4) calves fed EFE-treated wheat straw and probiotic supplement (EFE+ Pro+). Exogenous fibrolytic enzymes were applied to wheat straw. Data are presented as means ± SEM.

**Figure 2:**
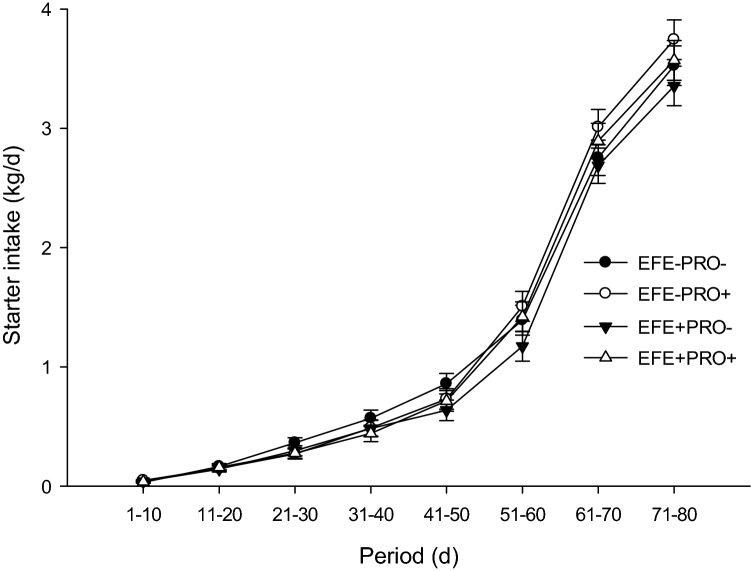
Starter feed intake in calves receiving different experimental diets. Treatments were (1) calves fed diets without exogenous fibrolytic enzymes (EFE)-treated wheat straw and probiotic supplement (EFE-Pro−), (2) calves fed diets of EFE-treated wheat straw but with probiotic supplement (2 g/d/calf; EFE-Pro+), (3) calves fed EFE-treated wheat straw (2 g/d/calf) but without probiotic supplement (EFE+ Pro−), (4) calves fed EFE-treated wheat straw and probiotic supplement (EFE+ Pro+). Exogenous fibrolytic enzymes were applied to wheat straw. Data are presented as means ± SEM.

**Figure 3:**
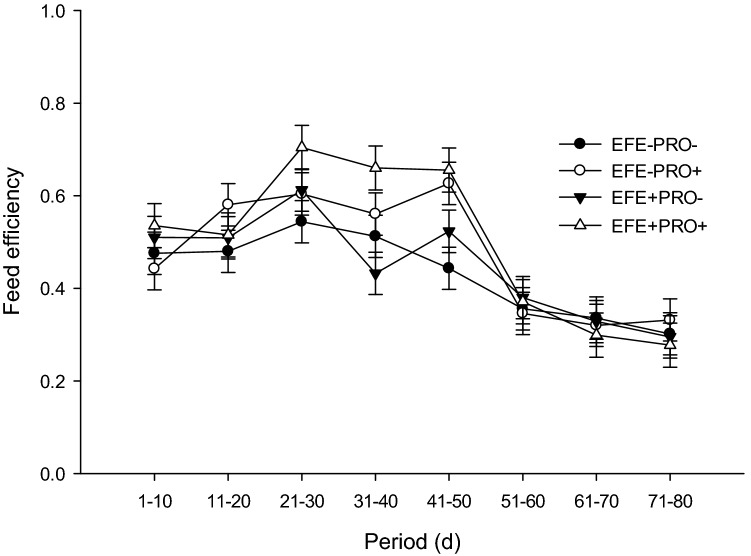
Feed efficiency in calves receiving different experimental diets. Treatments were (1) calves fed diets without exogenous fibrolytic enzymes (EFE)-treated wheat straw and probiotic supplement (EFE-Pro−), (2) calves fed diets of EFE-treated wheat straw but with probiotic supplement (2 g/d/calf; EFE-Pro+), (3) calves fed EFE-treated wheat straw (2 g/d/calf) but without probiotic supplement (EFE+ Pro−), (4) calves fed EFE-treated wheat straw and probiotic supplement (EFE+ Pro+). Exogenous fibrolytic enzymes were applied to wheat straw. Data are presented as means ± SEM.

Calf health-related data are presented in Table 2. No effect of EFE-treated wheat straw in the starter diet was observed on starter feed intake, NFC intake, BW, ADG, and FE. The probiotic supplement had no effect on starter feed intake or NFC intake. Calves fed a probiotic supplement had higher total ADG (*P* < 0.01), preweaning BW, total BW, FE (*P* < 0.01), weaning weight, and final weight (*P* < 0.01) than calves fed a non-supplemented diet. We observed no interaction between EFE-treated wheat straw and probiotic supplementation for starter feed intake, NFC intake, BW, ADG, and FE. A 2-way interaction was observed between probiotic supplementation and time to BW (Fig. 1, *P* < 0.01), starter feed intake (Fig. 2, *P* < 0.01), and FE (Fig. 3, *P* < 0.01), suggesting that probiotic supplementation had a greater effect on starter feed intake and ADG during the postweaning period and on FE during the preweaning period.

**Table 2:** Least squares means for starter feed intake, NFC intake, ADG, body weight (BW), and feed efficiency in dairy calves supplemented with or without exogenous fibrolytic enzymes (EFE) and probiotic (PRO) in the starter diets (n = 12 calves per treatment).

Item	EFE−		EFE+		SEM	*P*-value						
	PRO−	Pro+	PRO−	Pro+		EFE	PRO	EFE × PRO	Time (T)	EFE × T	PRO × T	PRO × EFE × T
**Starter feed intake**												
Pre-weaning	0.562	0.531	0.461	0.507	0.051	0.23	0.88	0.45				
Post-weaning	3.139	3.377	3.021	3.232	0.146	0.37	0.13	0.92				
Overall	1.206	1.243	1.101	1.188	0.067	0.28	0.41	0.73	< .0001	0.88	< 0.01	0.54
**NFC intake, kg/d**												
Pre-weaning	0.277	0.266	0.245	0.269	0.026	0.57	0.82	0.50				
Post-weaning	1.551	1.692	1.607	1.704	0.075	0.64	0.12	0.77				
Overall	0.596	0.622	0.586	0.627	0.037	0.97	0.37	0.84	< .0001	0.83	0.20	0.81
**ADG**												
Pre-weaning	0.571	0.636	0.563	0.676	0.028	0.57	0.002	0.41				
Post-weaning	0.995	1.085	0.939	0.904	0.058	0.05	0.64	0.30				
Overall	0.677	0.748	0.657	0.733	0.027	0.52	0.01	0.93	< .0001	0.19	0.14	0.48
**BW**												
Pre-weaning	56.8	58.4	56.7	59.8	1.05	0.55	0.04	0.51				
Post-weaning	88.9	93.7	87.6	93.5	1.85	0.69	0.01	0.78				
Overall	64.8	67.3	64.4	68.2	1.22	0.82	0.02	0.60	< .0001	0.24	0.001	0.60
Weaning	74.1	78.0	73.7	80.3	1.70	0.62	0.01	0.96				
Final	94.2	99.8	92.5	98.2	2.25	0.44	0.01	0.97				
**Feed efficiency**												
Pre-weaning	0.468	0.526	0.494	0.573	0.024	0.14	0.001	0.67				
Post-weaning	0.318	0.325	0.311	0.288	0.020	0.29	0.69	0.47				
Overall	0.431	0.476	0.448	0.502	0.019	0.25	0.01	0.83	< .0001	0.46	0.04	0.46

Treatments were (1) calves fed diets without exogenous fibrolytic enzymes (EFE)-treated wheat straw and probiotic supplement (EFE-Pro−), (2) calves fed diets of EFE-treated wheat straw but with probiotic supplement (2 g/d/calf; EFE-Pro+), (3) calves fed EFE-treated wheat straw (2 g/d/calf) but without probiotic supplement (EFE+ Pro−), (4) calves fed EFE-treated wheat straw and probiotic supplement (EFE+ Pro+). Exogenous fibrolytic enzymes were applied to wheat straw.

Body measurements data are presented in Table 3. No effect of EFE-treated wheat straw in the starter feed intake was observed on hip height and hip width. Calves fed EFE-treated wheat straw in the starter diet had lower body length (trend, *P* = 0.07), heart girth at weaning, and body barrel (*P* < 0.01) than calves fed untreated wheat straw in the starter diet. The probiotic supplement had no effect on heart girth, body barrel, or hip height. Calves fed a probiotic supplement had greater body length and hip width at weaning (*P* < 0.05) than calves fed a non-supplemented diet. We observed no interaction in body measurements between EFE-treated wheat straw and probiotic supplementation. A 2-way interaction was observed between probiotic supplementation and time to hip width (*P* = 0.03), suggesting that probiotic supplementation affected hip width only during the period before weaning. A 2-way interaction was observed between EFE-treated wheat straw in the starter diet and time to heart girth (*P* < 0.01), suggesting that EFE-treated wheat straw affects heart girth only during the preweaning period.

**Table 3:** Least squares means for body measurements in dairy calves supplemented with or without exogenous fibrolytic enzymes (EFE) and probiotic (PRO) in the starter diets (n = 12 calves per treatment).

Item	EFE−		EFE+		SEM	*P*-value						
	PRO−	Pro+	PRO−	Pro+		EFE	PRO	EFE × PRO	Time (T)	EFE × T	PRO × T	PRO × EFE × T
**Body length (cm)**												
Weaning	48.0	48.3	47.3	48.1	0.324	0.53	0.46	0.82				
Final	51.7	53.6	51.8	51.9	0.479	0.07	0.04	0.07				
Overall	49.9	50.9	49.7	50.0	0.413	0.16	0.10	0.35	< 0.01	0.28	0.26	0.06
**Heart girth (cm)**												
Weaning	103.4	103.2	100.5	99.5	0.96	0.001	0.48	0.70				
Final	108.3	107.4	108.2	106.7	0.79	0.34	0.16	0.73				
Overall	105.9	105.3	104.4	103.2	0.802	0.02	0.27	0.70	< 0.01	< 0.01	0.47	0.96
**Body barrel (cm)**												
Weaning	111.4	111.1	107.3	108.6	1.085	0.004	0.62	0.47				
Final	123.1	123.2	120.6	119.3	1.205	0.01	0.66	0.59				
Overall	117.3	117.1	113.9	113.9	1.09	0.004	0.97	0.93	< 0.01	0.96	0.18	0.10
**Hip height (cm)**												
Weaning	86.6	86.8	86.5	85.5	0.51	0.18	0.40	0.20				
Final	90.0	90.4	91.5	90.3	0.750	0.32	0.51	0.24				
Overall	88.3	88.62	89.1	87.8	0.48	0.98	0.31	0.13	< 0.01	0.06	0.64	0.60
**Hip width (cm)**												
Weaning	19.2	19.2	19.0	19.9	0.233	0.76	0.05	0.25				
Final	21.3	21.5	21.9	21.7	0.312	0.18	0.87	0.39				
Overall	20.3	20.4	20.5	20.7	0.23	0.27	0.42	0.85	< 0.01	0.08	0.03	0.08

Treatments were (1) calves fed diets without exogenous fibrolytic enzymes (EFE)-treated wheat straw and probiotic supplement (EFE-Pro−), (2) calves fed diets of EFE-treated wheat straw but with probiotic supplement (2 g/d/calf; EFE-Pro+), (3) calves fed EFE-treated wheat straw (2 g/d/calf) but without probiotic supplement (EFE+ Pro−), (4) calves fed EFE-treated wheat straw and probiotic supplement (EFE+ Pro+). Exogenous fibrolytic enzymes were applied to wheat straw.

### Health status

There were no effects of EFE-treated wheat straw and probiotic supplementation and their interactions on fecal score ≥ 2, rectal temperature > 39.2, electrolyte treatment, or medication day (Table 4).

**Table 4:** Least squares means for health status in dairy calves supplemented with or without exogenous fibrolytic enzymes (EFE) and probiotic (PRO) in the starter diets (n = 12 calves per treatment).

Item	EFE−		EFE+		SEM	*P*-value		
	PRO−	PRO+	PRO−	PRO+		EFE	PRO	EFE × PRO
Fecal score ≥ 2	4.72	3.33	3.33	3.54	0.942	0.48	0.48	0.34
Rectal temperature > 39.2	2.00	1.50	1.41	2.45	0.475	0.70	0.57	0.11
Electrolyte treatment	3.50	3.16	2.91	3.09	0.679	0.63	0.90	0.71
Medication day	1.16	0.75	0.83	0.18	0.302	0.14	0.08	0.70

Treatments were (1) calves fed diets without exogenous fibrolytic enzymes (EFE)-treated wheat straw and probiotic supplement (EFE-Pro−), (2) calves fed diets of EFE-treated wheat straw but with probiotic supplement (2 g/d/calf; EFE-Pro+), (3) calves fed EFE-treated wheat straw (2 g/d/calf) but without probiotic supplement (EFE+ Pro−), (4) calves fed EFE-treated wheat straw and probiotic supplement (EFE+ Pro+). Exogenous fibrolytic enzymes were applied to wheat straw.

### Behavioral data

Data on calf behavior are presented in Table 5. The addition of probiotics in the starter feed had no effect on the time calves spent standing, lying, eating, and NNOB. No effect of EFE-treated wheat straw in the starter feed was observed on time spent drinking and rumination. The EFE-treated wheat straw and the probiotic supplement had an interaction effect on standing time and NNOB time, with calves fed EFE+  PRO− having more standing time and less NNOB time compared to the other groups.

**Table 5:** Least squares means for behavior in dairy calves supplemented with or without exogenous fibrolytic enzymes (EFE) and probiotic (PRO) in the starter diets (n = 12 calves per treatment).

Item	EFE−		EFE+		SEM	*P*-value		
	PRO−	PRO+	PRO−	PRO+		EFE	PRO	EFE × PRO
Standing (min)	114.9	120.6	124.7	112.6	3.77	0.82	0.39	0.02
Lying (min)	179.0	181.3	188.4	189.6	4.31	0.04	0.68	0.89
Eating (min)	47.7	45.2	43.7	43.1	3.51	0.38	0.66	0.76
Drinking (min)	15.7	16.0	16.3	17.0	0.65	0.22	0.42	0.70
Ruminating (min)	75.7	71.2	70.3	75.5	2.95	0.49	0.69	0.27
NNOB (min)	46.8	45.5	36.5	45.1	2.45	0.03	0.14	0.04

Treatments were (1) calves fed diets without exogenous fibrolytic enzymes (EFE)-treated wheat straw and probiotic supplement (EFE-Pro−), (2) calves fed diets of EFE-treated wheat straw but with probiotic supplement (2 g/d/calf; EFE-Pro+), (3) calves fed EFE-treated wheat straw (2 g/d/calf) but without probiotic supplement (EFE+ Pro−), (4) calves fed EFE-treated wheat straw and probiotic supplement (EFE+ Pro+). Exogenous fibrolytic enzymes were applied to wheat straw.

*NNOB* non-nutritive oral behavior.

### Digestibility and ruminal fermentation

Nutrient digestibility data are presented in Table 6. The addition of probiotics and EFE-treated wheat straw to the starter feed did not affect the digestibility of DM, EE, and CP. NDF digestibility was improved by both EFE (*P* = 0.04) and the supplementation of probiotics (*P* = 0.001). Wheat straw treated with EFE and the probiotic supplement had an interaction effect on ADF digestibility, with calves fed EFE-PRO having lower ADF digestibility compared with the other groups.

**Table 6:** Least squares means for digestibility in dairy calves supplemented with or without exogenous fibrolytic enzymes (EFE) and probiotic (PRO) in the starter diets (n = 12 calves per treatment).

Parameters	Treatments				SEM	*P*-value		
	EFE-		EFE+					
	PRO−	PRO+	PRO−	PRO+		EFE	PRO	EFE × PRO
**Digestibility, %**								
Dry matter	75.4	77.8	77.1	76.9	0.67	0.52	0.11	0.07
Ether extract	80.9	82.9	82.6	82.7	0.98	0.44	0.29	0.34
Crude protein	71.5	73.3	73.9	73.0	0.92	0.26	0.64	0.13
Neutral detergent fiber	42.2	48.9	46.1	52.3	1.70	0.03	0.001	0.89
Acid detergent fiber	38.3	45.8	46.1	44.7	2.30	0.14	0.18	0.05

Treatments were (1) calves fed diets without exogenous fibrolytic enzymes (EFE)-treated wheat straw and probiotic supplement (EFE-Pro−), (2) calves fed diets of EFE-treated wheat straw but with probiotic supplement (2 g/d/calf; EFE-Pro+), (3) calves fed EFE-treated wheat straw (2 g/d/calf) but without probiotic supplement (EFE+ Pro−), (4) calves fed EFE-treated wheat straw and probiotic supplement (EFE+ Pro+). Exogenous fibrolytic enzymes were applied to wheat straw.

Ruminal fermentation profile data are presented in Table 7. We observed no interaction between EFE-treated wheat straw and probiotic supplementation on rumen fermentation parameters. No effect of EFE-treated wheat straw was observed on rumen pH, total SCFA, and molar proportions of propionate, butyrate, iso-butyrate, valerate, and iso-valerate in the rumen. Calves fed EFE-treated wheat straw in the starter diet had lower molar proportions of acetate in the rumen (d 70 *P* = 0.05; overall *P* = 0.09) and a lower ratio of acetate to propionate (d 70 *P* = 0.03) than calves fed untreated wheat straw in the starter diet. The probiotic supplement did not affect the total amount of SCFA in the rumen or the molar proportions of butyrate, isobutyrate, valerate, and iso-valerate in the rumen. Calves fed a probiotic supplement had greater molar proportions of propionate in the rumen (*P* < 0.05) and less acetate (*P* < 0.05) on day 35 of the study than calves fed no supplement.

**Table 7:** Least squares means for ruminal fermentation in dairy calves supplemented with or without exogenous fibrolytic enzymes (EFE) and probiotic (PRO) in the starter diets (n = 12 calves per treatment).

Item	EFE-		EFE+		SEM	*P*-value						
	PRO−	PRO+	PRO−	PRO+		EFE	PRO	EFE × PRO	Time (T)	EFE × T	PRO × T	PRO × EFE × T
**Total SCFA, mmol/L**												
d 35	85.2	84.4	86.2	71.4	5.27	0.37	0.25	0.29				
d 70	141.5	146.1	150.2	144.4	9.26	0.70	0.94	0.57				
Overall	113.4	115.3	118.2	107.9	5.81	0.83	0.47	0.29	< 0.01	0.40	0.52	0.87
**SCFA, mol/100 mol**												
Acetate												
d 35	54.2	52.1	55.7	49.0	1.85	0.67	0.02	0.21				
d 70	49.1	49.0	46.8	46.4	1.22	0.04	0.83	0.90				
Overall	51.7	50.5	51.2	47.7	0.97	0.09	0.02	0.22	< 0.01	0.48	0.09	0.39
**Propionate**												
d 35	34.9	36.8	33.4	39.8	1.81	0.69	0.02	0.21				
d 70	40.3	42.2	42.7	44.7	1.76	0.17	0.26	0.98				
Overall	37.6	39.5	38.0	42.2	1.25	0.15	< 0.01	0.30	< 0.01	0.54	0.44	0.43
**Butyrate**												
d 35	7.74	6.84	7.84	6.71	1.044	0.98	0.33	0.90				
d 70	7.41	6.19	8.01	6.94	0.94	0.47	0.23	0.93				
Overall	7.58	6.52	7.93	6.83	0.69	0.63	0.12	0.97	0.84	0.62	0.92	0.89
**Iso-butyrate**												
d 35	0.42	0.72	0.37	0.55	0.178	0.54	0.19	0.74				
d 70	0.23	0.25	0.30	0.19	0.06	0.92	0.52	0.35				
Overall	0.33	0.48	0.34	0.37	0.09	0.59	0.32	0.53	< 0.01	0.54	0.15	0.97
**Iso-valerate**												
d 35	0.65	1.23	0.74	0.97	0.28	0.76	0.15	0.52				
d 70	0.49	0.41	0.56	0.35	0.125	0.93	0.24	0.58				
Overall	0.57	0.82	0.65	0.66	0.173	0.83	0.47	0.48	< 0.01	0.70	0.03	0.67
**Valerate**												
d 35	1.98	2.32	1.93	2.91	0.45	0.55	0.14	0.48				
d 70	2.42	1.83	2.09	2.10	0.337	0.93	0.39	0.38				
Overall	2.20	2.08	2.01	2.51	0.215	0.57	0.38	0.15	0.60	0.66	0.16	0.97
**Acetate: propionate**												
d 35	1.67	1.43	1.69	1.25	0.102	0.45	< 0.01	0.32				
d 70	1.32	1.16	1.10	1.04	0.076	0.03	0.15	0.56				
Overall	1.49	1.30	1.40	1.14	0.051	0.21	< 0.01	0.57	< 0.01	0.53	0.12	0.32

Treatments were (1) calves fed diets without exogenous fibrolytic enzymes (EFE)-treated wheat straw and probiotic supplement (EFE-Pro−), (2) calves fed diets of EFE-treated wheat straw but with probiotic supplement (2 g/d/calf; EFE-Pro+), (3) calves fed EFE-treated wheat straw (2 g/d/calf) but without probiotic supplement (EFE+ Pro−), (4) calves fed EFE-treated wheat straw and probiotic supplement (EFE+ Pro+). Exogenous fibrolytic enzymes were applied to wheat straw.

### PCA

**Figure 4:**
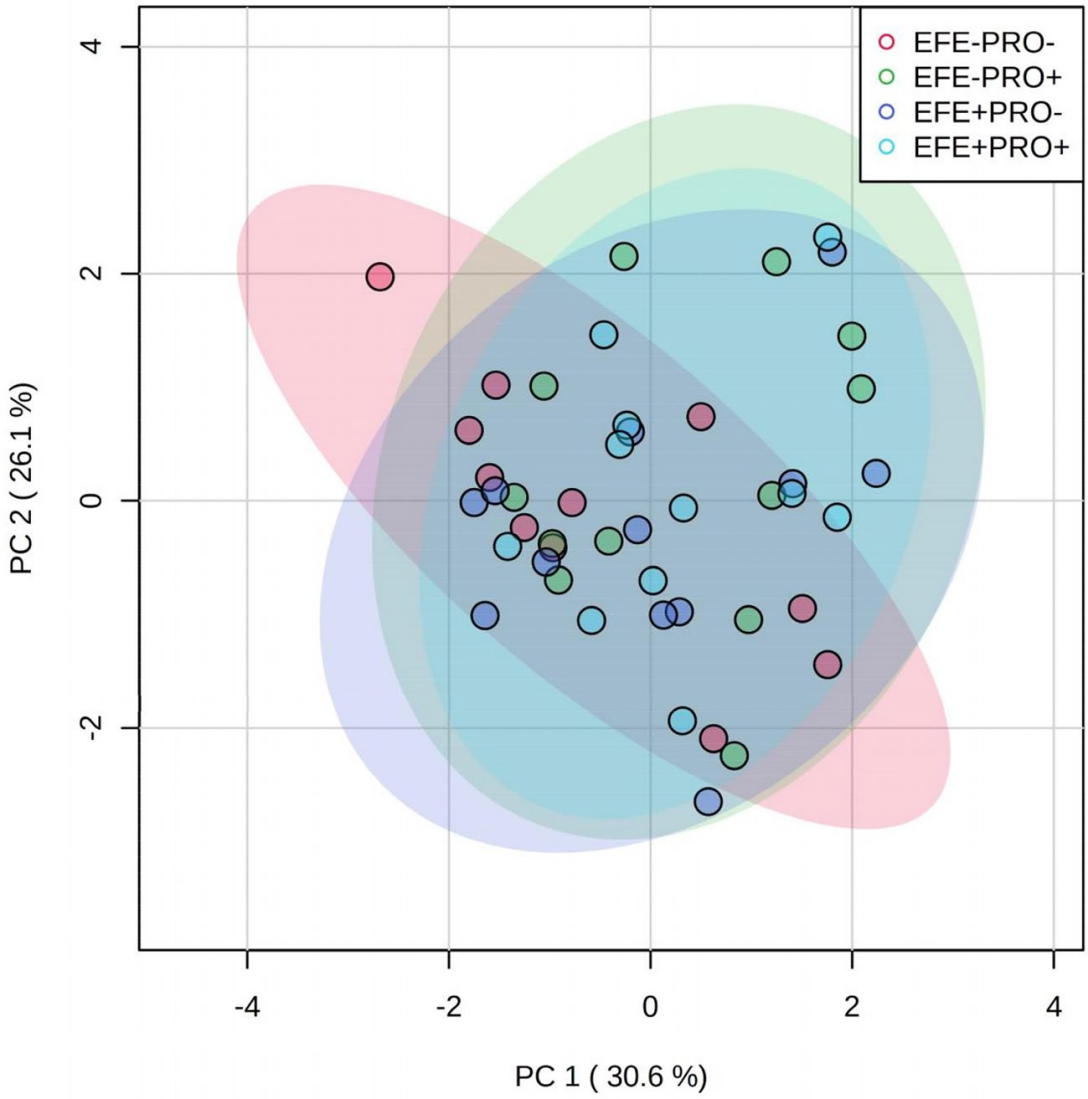
Principal component analysis (PCA) shows the interaction of the different experimental diets (colored) for all log-transformed and Pareto-scaled data for growth performance, health, and behavior. Treatments were (1) calves fed diets without exogenous fibrolytic enzymes (EFE)-treated wheat straw and probiotic supplement (EFE-Pro−), (2) calves fed diets of EFE-treated wheat straw but with probiotic supplement (2 g/d/calf; EFE-Pro+), (3) calves fed EFE-treated wheat straw (2 g/d/calf) but without probiotic supplement (EFE+ Pro−), (4) calves fed EFE-treated wheat straw and probiotic supplement (EFE+ Pro+). Exogenous fibrolytic enzymes were applied to wheat straw.

 For the growth performance, health, and behavior data, PCA revealed two principal components (PC1 and PC2) that explained 56% of the variance, and even the treatment groups overlapped and could not be separated (Fig. 4).

## Discussion

### Fibrolytic enzyme effects

In the present study, the dietary NFC concentration increased when wheat straw was treated with EFE, which could be explained by the partial hydrolysis of the hemicellulose but not the cellulose fraction to monosaccharides and oligosaccharides by the activity of fibrolytic enzymes^34^. In agreement with our findings, Rodrigues et al.^35^ reported that pretreatment of wheat straw with enzyme extracts isolated from white rot fungi with ligninolytic and cellulolytic activities degraded hemicellulose content, resulting in lower NDF concentration^35^. They indicated that hemicellulose was preferentially degraded by the enzymatic extracts. Fibrolytic enzymes pretreating the forage can weaken and/or degrade the lignin-hemicellulose-cellulose complex of the plant^36^. This loosening effect could facilitate the attachment of microorganisms to the substrate, leading to faster growth of microbial populations and increased fiber degradation^37,38^. In addition, it has been reported that exogenous enzymes can synergistically enhance the hydrolytic potential of endogenous microbial enzymes in the rumen, resulting in improved digestion of dietary fiber in the rumen^39^, which is consistent with the higher NDF digestion when the calves in the current study received EFE-treated wheat straw. In addition, the higher NDF digestibility with enzyme pretreatment may be due to the relatively prolonged retention time of the feed in the rumen caused by the lower feed intake during the study. The prolonged retention time of fiber in the rumen could improve fiber digestion by EFE administration.

Feeding EFE-treated wheat straw decreased starter feed intake before weaning and throughout the study, but had no effect on calf NFC intake, ADG, BW, or feed efficiency. To our knowledge, there is no previous report on pretreatment of low-quality forages such as straw with fibrolytic enzymes for feeding newborn dairy calves. Studies on pretreatment of forages with fibrolytic enzymes on feed intake have shown conflicting results. Some researchers reported that EFE supplementation had no effect on feed intake of growing buffaloes^12^ and weaned dairy calves^40^. Wang et al.^11^ showed that supplementation with fibrolytic enzymes tended to increase feed intake of bull calves aged 46 to 90 days. The discrepancy between our results and those of other researchers could be due to the method of administration of fibrolytic enzymes (direct administration into the starter feed versus pretreatment of the forage with fibrolytic enzymes), the composition of the basal feed, especially fiber sources or fiber content, which affect the activity and stability of rumen enzymes^14^. The lower concentration of NDF and higher digestibility of NDF should improve feed intake. Although the exact mechanism is not clear, the higher digestibility of forages pretreated with enzymes could result in greater amounts of oxidizable fuels being available to hepatocytes, thereby reducing feed intake.

In the current study, lower intake of starter feed and ME was not associated with poorer ADG or feed efficiency because lower feed intake was compensated by higher fiber digestibility, resulting in similar growth performance. However, skeletal growth parameters, including final body length, heart girth at weaning, and body barrel at weaning and finishing were lower in calves fed EFE-treated wheat straw. These results suggest that enzyme treatment of straw limited the supply of nutrients other than energy needed for skeletal growth. Furthermore, in addition to improved fiber digestion in calves fed EFE, lower feed intake could be related to lower intestinal filling^41^, resulting in lower body barrel as an indicator of intestinal filling^42^ in calves.

Feeding diets containing EFE-treated wheat straw had no effect on rumen fermentation parameters, except that the molar proportion of acetate and the ratio of acetate to propionate in the rumen decreased with EFE supplementation. It had been noted earlier that NFC concentrations were higher in diets containing EFE-treated wheat straw, which may have contributed to a shift in rumen fermentation toward more propionogenesis, as indicated by the numerically higher molar proportion of propionate production at the expense of acetate, resulting in a lower acetate-to-propionate ratio. In agreement with our results, Ribeiro et al.^43^ reported that treatment with a recombinant fibrolytic enzyme 24 h after feeding decreased the molar ratio of acetate, which was consistent with a lower protozoan population in the rumen. As a result of fermentation of starch and cellulose by protozoa, acetate and butyrate are the main end products^44^.

### Probiotic supplementation effects

Probiotic products generally contain a mixture of microorganisms, including *S. cerevisiae* and lactic acid bacteria, that optimize the intestinal health of animals by competing with undesirable microorganisms, stimulating the production of beneficial bacteria, and increasing nutrient absorption in the gut^45,46^. The effect of feed supplements such as probiotics has also been shown to depend on the type of supplementation, with solid feed supplementation having a more pronounced effect^47–49^. However, in the first weeks of life, when calves are exposed to stressful situations, solid feed intake is not sufficient to provide calves with adequate feed supplements. Interestingly, our results showed that probiotic addition improved the uptake of the starter feed around weaning but not throughout the study period, which may suggest that probiotic addition had an ameliorative effect on the weaning stress. Chaucheyras-Durand et al.^50^ indicated that yeast products are particularly useful during periods of dietary change and stress. Milk restriction before weaning causes calves to consume significant amounts of solid feed, which can increase acidity in the rumen and lead to a decrease in rumen pH and eventually rumen acidosis^51,52^. Silberberg et al.^53^ reported that during acidosis challenges, yeast supplementation stabilized fermentative parameters, promoted protozoal numbers, decreased lactate-producing bacteria, and helped normalize the animals' inflammatory status. In addition, probiotic supplementation improved NDF digestibility, which would stimulate the intake of DM. Consistent with our results, some studies have found that starter diets increased when claves were supplemented with yeast fermentation products in the starter diet^47,54^.

In addition, probiotic supplementation improved preweaning ADG and total ADG, BW, and feed efficiency in the current study. In general, the improvement in growth performance in the current study might be related to the improved rumen fermentation, nutrient digestibility, and health status of the probiotic-supplemented calves. Consistent with our findings, intensive research has reported that growth performance was improved when young beef or dairy calves were supplemented with active dry yeast^55,56^, yeast fermentation products^47,48^, or *Bacillus amyloliquefaciens strain H57*^19^ compared to control calves. In addition, calves supplemented with probiotics exhibited higher feed efficiency, which may be associated with greater fiber digestion in the rumen and improved rumen fermentation, as indicated by the lower acetate-propionate ratio without affecting total SCFA concentration, which may improve growth performance. The lower acetate-propionate ratio provides faster and more energy to the body, which has a positive effect on animal growth performance^56^. In general, a higher acetate-propionate ratio means better digestibility of cellulose^57^. However, the results of the current study suggest that improved fiber digestion was accompanied by a shift in rumen fermentation toward propionate synthesis at the expense of acetate production. Consistent with our results, administration of yeast to weaned beef calves^56^ or *Lactobacillus rhamnosus GG* to newborn calves improved NDF digestion and promoted propionate production^30^. Probiotics, including yeast cultures or direct feeding bacterial microbiome, are thought to improve animal performance by influencing the rumen microbiota to support the growth and activity of fibrolytic microorganisms and those that metabolize lactate^58,59^. Therefore, probiotic administration in the current study could promote the growth and metabolism of lactate-utilizing bacteria in the rumen and then support the conversion of lactate to propionate, allowing calves to obtain more energy from their diet^60^. Consistent with our results, dietary supplementation with *Bacillus amyloliquefaciens H57* improved feed conversion of calves by 16.2% compared to calves without supplementation^19^.

Calf health is a critical factor affecting welfare and growth performance during early life and future productivity^61^. Probiotics can have beneficial effects on gastrointestinal functions in humans and animals and other health-related outcomes. It has been reported that the inclusion of yeast-based^47,48,62,63^ or bacterial-based^19^ live probiotics in calf starter feed improved fecal scores and some other health-related indicators in young calves. The administration of yeast-based probiotics has been shown to reduce diarrhea by modulating the composition of the intestinal microbiome, preventing the binding of pathogenic bacteria to intestinal epithelial cells, modulating intestinal mucosa and systemic immunity, improving the integrity of the intestinal barrier, and reducing the infiltration of toxic luminal antigens and bacteria^15^. However, probiotic supplementation did not improve the health status of the dairy calves in the current study.

## Conclusion

The results of the study showed that wheat straw treated with EFE had no positive effects on starter feed intake, performance, and health status of calves during the preweaning and postweaning periods. Nevertheless, supplementing calf diets with wheat straw treated with EFE has the potential to improve NDF digestibility. The diet supplemented with probiotics showed a potential improvement in feed efficiency and growth performance of dairy calves, as evidenced by increased ADG and final weight. The addition of probiotics increased the digestion of NDF and altered rumen fermentation. Probiotic administration to calves requires further research to better understand the underlying mechanisms responsible for the observed improvement in feed efficiency and growth performance so that better supplementation strategies can be implemented. Given the limited data, future studies need to investigate whether probiotics should be used prophylactically or therapeutically in young calves.

## Supplementary Information

Supplementary Information.

## Data Availability

All data generated or analysed during this study are included in this published article [and its supplementary information files].
